# In-office tooth bleaching with 38% hydrogen peroxide promotes moderate/severe pulp inflammation and production of *ll-1β, TNF-β, GPX, FGF-2* and osteocalcin in rats

**DOI:** 10.1590/1678-7757-2017-0367

**Published:** 2018-05-22

**Authors:** Renata Suellen Galvão da Silva-Costa, Andressa Eveline de Lima Ribeiro, Isauremi Vieira de Assunção, Raimundo Fernandes de Araújo, Aurigena Antunes de Araújo, Gerlane Coelho Bernardo Guerra, Boniek Castillo Dutra Borges

**Affiliations:** 1 Universidade Federal do Rio Grande do Norte, Departamento de Odontologia, Programa de Pós-Graduação em Saúde Pública, Natal, Rio Grande do Norte, Brasil.; 2 Universidade Federal do Rio Grande do Norte, Departamento de Morfologia, Programa de Pós-Graduação em Ciências da Saúde, Programa de Pós-Graduação em Biologia Funcional e Estrutural, Natal, Rio Grande do Norte, Brasil.; 3 Universidade Federal do Rio Grande do Norte, Departamento de Biofísica e Farmacologia, Programa de Pós-Graduação em Ciências Farmacêuticas, Programa de Pós-Graduação em Saúde Pública, Natal, Rio Grande do Norte, Brasil.; 4 Universidade Federal do Rio Grande do Norte, Departamento de Biofísica e Farmacologia, Programa de Pós-Graduação em Ciências Biológicas, Programa de Pós-Graduação em Ciências Farmacêuticas, Natal, Rio Grande do Norte, Brasil.

**Keywords:** Dental pulp, Inflammation mediators, Tooth bleaching

## Abstract

**Objectives:**

To study the intensity of inflammatory infiltrate and production of interleukin-1β (ll-1β), tumor necrosis factor-β (*TNF-β*), fibroblast growth factor-2 (*FGF-2*), glutathione peroxidase (*GPX*), and osteocalcin in response to in-office tooth bleaching in rats.

**Material and Methods:**

Twenty male Wistar rats were randomized into four groups (n=5) according to the received treatment (tooth bleaching or no treatment - control) and the period of euthanasia after treatment (24 h or 10 days). We performed tooth bleaching using a 38% hydrogen peroxide gel on maxillary and mandibular incisors. After euthanasia, incisors (20 *per* group) were processed for histological analysis, immunohistochemistry staining of *ll-1β*, *TNF-β*, *FGF-2* and *GPX* and osteocalcin by immunofluorescence. We analyzed data using the Mann-Whitney and Kruskal-Wallis/Dunn tests (p<0.05).

**Results:**

The bleached groups presented statistically significant differences regarding the pulp inflammation stage compared with the control groups. Bleached teeth showed moderate/severe inflammatory infiltrate and control groups presented absent inflammatory cells or a negligible number of mononuclear cells (p<0.001) at two times (24 h and 10 days). There was strong staining for *ll-1β*, *TNF-β*, and *GPX* in bleached groups at 24 h and strong staining for *ll-1β*, *TNF-β*, *GPX* and *FGF-2* at 10 days. After 10 days of tooth bleaching, the bleached group showed a statistically superior amount of osteocalcin than the other groups (p<0.01).

**Conclusions:**

Tooth bleaching with 38% hydrogen peroxide causes severe pulp inflammation, but characteristics of tissue repair after 10 days.

## Introduction

Tooth bleaching has been widely used to correct tooth discoloration and thus producing a pleasing smile, since it is an effective and conservative approach to whiten stained teeth^12^. In-office or at-home tooth bleaching approaches may be performed by dental professionals^12^. In-office bleaching requires the application of highly concentrated hydrogen peroxide (HP) on dental enamel, being a practical alternative to at-home bleaching treatment, with severe discoloration, poor patient compliance, and rapid results^15^. This method has been around for many years and remains popular because results can be seen after one appointment^23^. However, in-office tooth bleaching may lead to side effects on dental tissues, such as pulp inflammation^5^ ^,^ ^26^. HP can penetrate the pulp chamber, leading to reversible inflammatory reactions in the pulp tissues because of chemical irritation^12^.

In-office tooth bleaching can lead to an increase of inflammatory cells, macrophage migration, some necrotic areas, congested large caliber blood vessels, and collagen degradation in the pulp^12^ ^,^ ^13^ ^,^ ^26^, which may be transitory, since dental pulp is capable of self-repair^17^. However, pro- and anti-inflammatory cytokines or chemical substances produced by the dental pulp in response to in-office tooth bleaching are not well-established in the literature; therefore, further understanding of bleaching-mediated pulp inflammation and repair processes is required.

Cytokines such as interleukin-1β (*ll-1β*) and tumor necrosis factor-β (*TNF-β*) are produced in dental pulpitis^11^ ^,^ ^18^ ^,^ ^22^. In contrast, fibroblast growth factor-2 (*FGF-2*)^19^, osteocalcin^1^, and glutathione peroxidase (*GPX*) enzymes are related to repair, regeneration, and antioxidant defense phenomena in dental pulp^2^ ^,^ ^10^. However, the production of these substances in dental pulp in response to in-office tooth bleaching is still unknown.

Tooth-bleaching procedure causes an inflammatory response, and our study aimed to analyze the intensity of the inflammatory process and the production of *ll-1β*, *TNF-β*, *FGF-2*, *GPX* and osteocalcin in tooth pulp after bleaching with 38% HP. The hypotheses were: (1) there will be a severe inflammatory process; and (2) production of *ll-1β*, *TNF-β*, *FGF-2*, *GPX* and osteocalcin.

## Material and methods

This experimental *in vivo* study was performed on 60-day-old male Wistar rats (180-220 g) housed under standard conditions (12 h light/dark cycle; 22±0.1°C) with access to food and water *ad libitum*. The animals were individually housed in autoclaved polypropylene cages measuring 41×34×16 cm. All animal protocols were approved by the Animal Ethics Committee (no. 69/2014) of the Federal University of Rio Grande do Norte, Brazil.

The sample size was based on previous studies^6^ ^,^ ^9^. Twenty Wistar rats were randomly divided into four groups according to treatment (tooth bleaching and no bleaching – control) and euthanasia period (24 h after tooth bleaching and 10 days after tooth bleaching). The upper and lower incisors of each animal were used for the same group, resulting in 80 analyzed teeth (n=20 *per* group). The animals in the two bleached groups (BG) received a bleaching procedure and were euthanized at different times (24 h and 10 days) after the last session. Animals from the two control groups (CG) were anesthetized, but tooth bleaching was not performed, and they were euthanized 24 h and 10 days after anesthesia, respectively.

Tooth bleaching was performed under anesthesia by intraperitoneal injection of 10% ketamine (80 mg/kg; Vetnil, São Paulo, SP, Brazil) and 2% xylazine (10 mg/kg; Calmium, São Paulo, SP, Brazil). A dentist performed all dental procedures. After application of a gingival barrier (FGM Dentscare LTDA, Joinville, SC, Brazil), 38% HP gel Opalescence Boost (Ultradent Products Inc., South Jordan, UT, USA) was handled according to the manufacturer’s protocol and applied on the labial surface of the incisors (0.02 ml *per* tooth). The treatment for the bleaching groups consisted of two applications of 15 min each with an exchange of gel between them, for 30-min exposure to the bleaching agent. The second bleaching session was performed 7 days after the first session. The animals in the control groups were anesthetized twice with a 7-day interval, but did not receive the bleaching agent.

After the procedures, animals were euthanized with 90 mg/kg thiopental (Cristália, São Paulo, SP, Brazil) at 24 h and 10 days after the last bleaching session (bleached group)/last anesthesia (control groups).

### Histopathologic analyses

The jaws from each rat were separated, dissected, and fixed in a solution of 10% buffered formalin for 24 h to obtain individual teeth. The tissues were then demineralized in 5% nitric acid for 15 days and dehydrated through a graded series of ethanol. The tissues were sectioned along the tooth direction for hematoxylin and eosin staining after they were embedded in paraffin. Sections of coronal pulp were evaluated by light microscopy Nikon E200 (Nikon Corporation; Tokyo, Japan). The representative areas for histopathological classification were chosen for analysis. The coronal pulp was classified according to the ascending order of inflammatory cell response and pulp tissue integrity based on previous study^9^: 0: Absent or negligible number of inflammatory cells; 1: Mild inflammatory infiltrate; 2: Moderate inflammatory infiltrate; and 3: Severe inflammatory infiltrate and/or necrosis. The histological analysis of scores was blindly conducted by two pathologists. Data were statistically analyzed by the Mann-Whitney test. The significance level was set at 95% (*p*<0.05).

### Immunohistochemistry staining of *ll-1β*, *TNF-β*, *FGF-2*, *GPX*

Three sections of a tooth (3 μm) were obtained with the use of a microtome from each group and transferred to gelatin-coated slides. Each tissue section was then deparaffinized and rehydrated. The tooth tissue slices were washed with 0.3% Triton X-100 in phosphate buffer and soaked with endogenous peroxidase (3% HP). Tissue sections were incubated overnight at 4°C with primary antibodies (Santa Cruz Biotechnology; Santa Cruz, CA, USA) against *ll-1β*, *TNF-β*, *FGF-2*, and *GPX*. Dilution tests (3 dilutions: 1:100; 1:400; 1:800) were performed with all antibodies. The dilution choices were: *ll-1β: 1:100*; *TNF-β: 1:400; FGF-2: 1:400;* and *GPX: 1:400.* Slices were washed with phosphate buffer and incubated with a streptavidin/HRP-conjugated secondary antibody (Dako; Carpinteria, CA, USA) for 30 min. Immunoreactivity to the antibodies was visualized with a colorimetric-based detection kit following the manufacturer’s protocol: Dako Liquid DAB + Substrate Chromogen System (Dako; Carpinteria, CA, USA). Planimetry microscopy with high-power magnification (40×) was used to visualize the intensity of immunostaining in the pulp tissue. The staining intensity was recorded as 1-weak or 2-strong^22^. Immunohistochemistry analyses were blindly conducted by two pathologists. Data were statistically analyzed by Mann-Whitney test. The significance level was set at 95% (*p*<0.05).

### Confocal immunofluorescence

The same immunohistochemistry protocol was used for confocal immunofluorescence, but the tissue sections were incubated overnight at 4°C with primary antibody (Santa Cruz Biotechnology, Santa Cruz, CA, USA) against osteocalcin 1:200 for 1 h. The tissues were washed in phosphate buffered saline/0.2% Triton X-100 for 5 min, and then were incubated with Alexa Fluor 488-conjugated goat anti-rabbit (Abcam Inc.; Cambridge, MA, USA) secondary antibody (1:500) for 1 h. We obtained fluorescent images using a Carl Zeiss laser scanning microscope - 20× objective (LSM 710; Carl Zeiss, Jena, Germany). Tissue reactivity in all groups was assessed by computerized densitometry analysis of the digital images captured with the confocal immunofluorescence microscope. Average densitometric values were calculated by ImageJ software (Wayne Rasband, National Institutes of Health; Bethesda, MD, USA). Data were statistically analyzed by the Kruskal-Wallis test followed by Dunn’s test. The significance level was set at 95% (*p*<0.05).

## Results

### Histopathologic analyses

**Figure 1:**
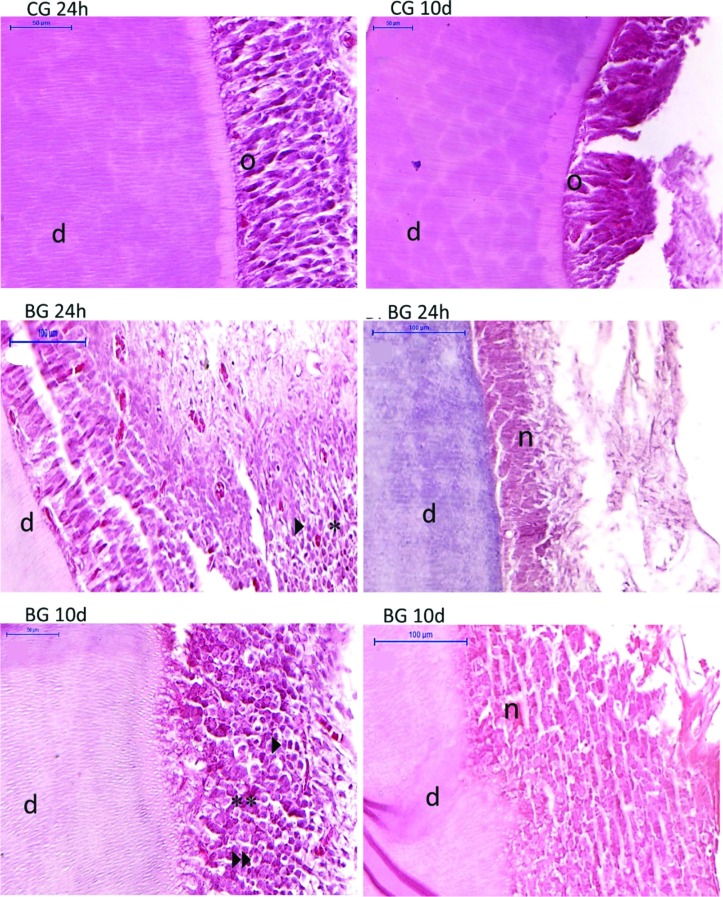
Histopathological analysis: CG 24h (control group 24 hours); CG 10d (control group 10 days); BG 24h (bleached group 24 hours); BG 10d (bleached group 10 days). d=dentin, n=necrosis; *= Mononuclear moderate inflammatory infiltrate; **= Intense inflammatory infiltrate; short arrow: lymphocyte: double arrow: macrophage

The control groups at 24 h (CG 24h) and 10 days (CG 10d) showed absent inflammatory cells or a negligible number of mononuclear cells. The bleached group at 24 h (BG 24h) showed areas of moderate inflammatory infiltrate, with prevalence of lymphocytes, and a focal area with moderate inflammatory infiltrate and necrosis. The bleached group at 10 days (BG10d) presented moderate inflammatory infiltrate with lymphocytes, macrophages, and extensive necrosis areas, as shown in Figure 1. There was a statistically significant difference between the CG groups and the BG groups (*p*<0.001) when the classification of each group for the inflammation stages was analyzed (Table 1).

**Table 1:** Histopathological and immunohistochemistry scores. Immunofluorescence analysis - mean (standard deviation)

Groups	Median (interquartile distance) for histopathological scores	Median (interquartile distance) for immunohistochemistry scores				Osteocalcin immunofluorescence mean (standard deviation)
		*Il-1β*	*TNF-β*	*FGF-2*	*GPX*	
CG 24 h	0 (0-0)	0 (0-0)	0 (0-0)	0 (0-0)	0 (0-0)	2.043 (0.2346)
BG 24 h	2 (1-2.5)*	1 (0.5-1)*	1 (0.5-1)*	0 (0-0)	1 (0.5-1)*	2.137 (0.6429)
CG 10 d	0 (0-0)	0 (0-0)	0 (0-0)	0 (0-0)	0 (0-0)	2.373 (0.6311)
BG 10 d	3 (2-2.5)#	1 (0.5-1)#	1 (0.5-1)#	1 (0.5-1)#	1 (0.5-1)#	7.767 (0.9592) ##

CG 24h (control group 24 hours); CG 10d (control group 10 days); BG 24h (bleached group 24 hours); BG 10d (bleached group 10 days)

* p<0.05 (Comparison between 24 h BG and 24 h CG groups)

# p<0.05 (Comparison between 10 d BG and 10 d CG groups)

## p<0.01 (Comparison between 10 d BG and 10 d CG groups)

### Immunohistochemical staining of *ll-1β*, *TNF-β*, *FGF-2*, *GPX*

**Figure 2:**
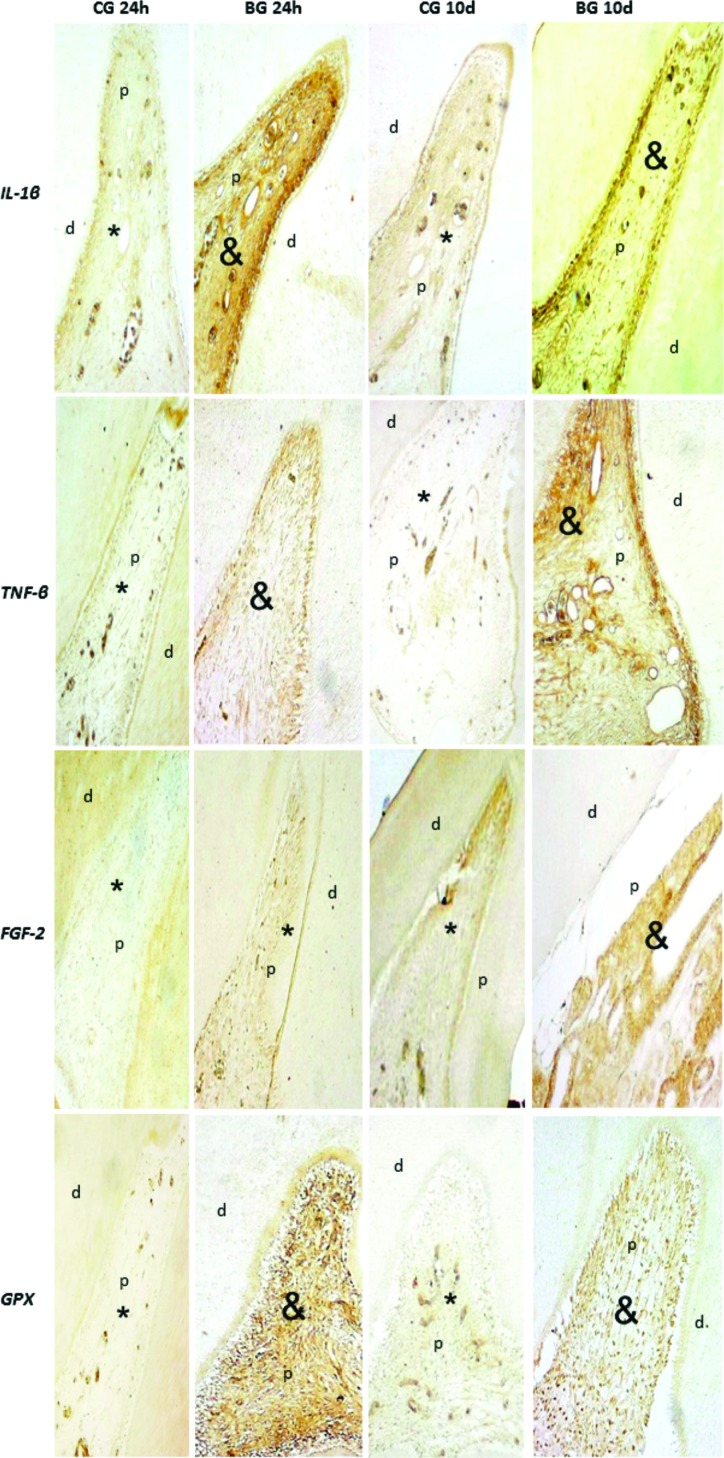
Immunohistochemistry analysis: CG 24h (control group 24 hours); CG 10d (control group 10 days); BG 24 h (bleached group 24 hours); BG 10d (bleached group 10 days). d=dentin, p=pulp. *Il-1β*; *TNF-β*; *FGF* and *GPX*. *Pulp presenting weak staining; & Pulp presenting strong staining

In the control groups of 24 hours (CG 24h) and 10 days (CG 10d) there was weak staining of *ll-1β*, *TNF-β*, *FGF-2* and *GPX*. In the bleached groups at 24 h (BG 24h) there was strong staining of *ll-1β*, *TNF-β* and *GPX* (*p*<0.05), and there was weak staining of *FGF-2* compared with CG 24h. In the bleached groups at 10 days (BG 10d) there was strong staining of *ll-1β*, *TNF-β*, *GPX* and *FGF-2* (*p*<0.05) compared with CG 10d, as shown in Figure 2 and Table 1.

### Confocal immunofluorescence

**Figure 3:**
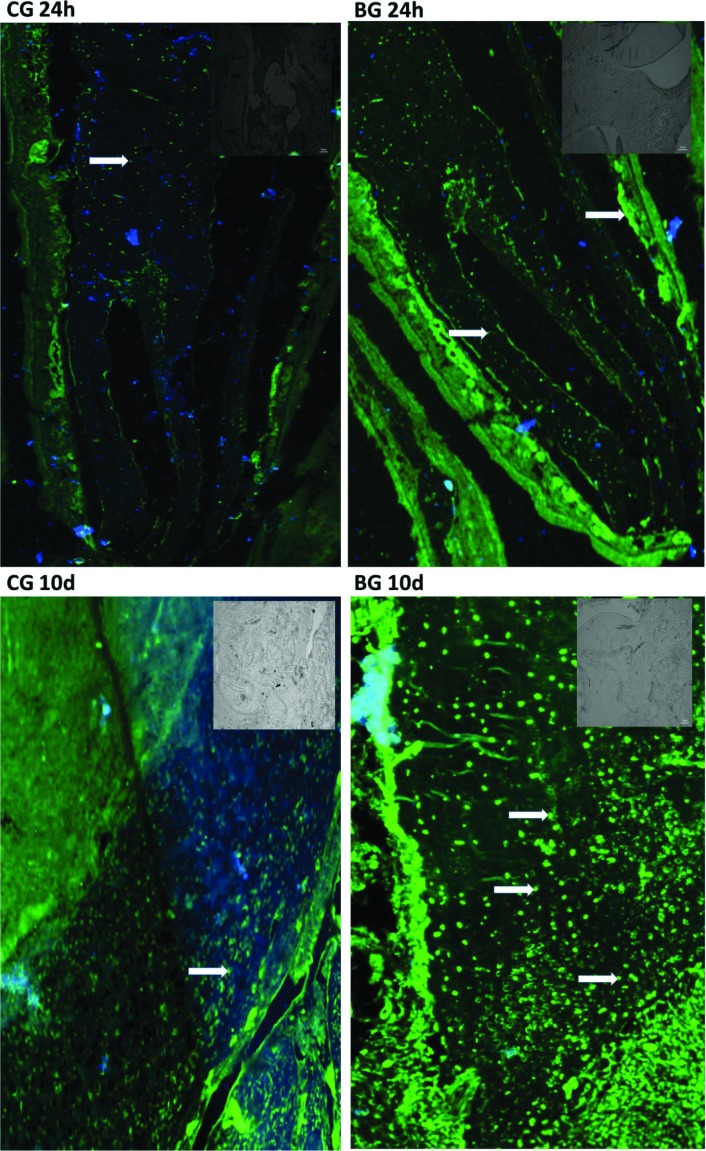
Confocal photomicrographs representative of immunoreactivity to osteocalcin: CG 24h (control group 24 hours); CG 10d (control group 10 days); BG 24h (bleached group 24 hours); BG 10d (bleached group 10 days). The fluorescent labeling of the Alexa-Fluor 488® secondary antibody directed to the primary anti-osteocalcin antibody is observed in green. Nuclear labeling of the inflammatory cells with DAPI is observed in blue. Arrow= osteocalcin; samples were counterstained with DAPI nuclear counterstained (blue). CG24h, BG 24h and CG 10d: Weak osteocalcin labelling (white arrows); BG10d: osteocalcin labelling (white narrow) was diffuse and strong. Scale bar, 100 mm, 10x. Small pictures - upper right: IgG controls

Increased osteocalcin production in the tissue 10 days after the clinical procedure is shown in Figure 3 by the stronger and diffuse green marking (Osteocalcin). A statistically higher value was found for the BG at 10 days compared with the BG at 24 h and the control group at 10 days (*p*<0.01) (Table 1).

## Discussion

Contact of hydrogen peroxide with the dental enamel causes mineral loss, thus increasing its porosity, which increases diffusion of the bleaching agent into deeper areas of dentin and pulp tissue^20^. By histologic examination, some studies have reported that depending on the type of pulp irritability and potency, minor alterations such as reversible inflammatory reactions or even tissue degeneration may occur^3^ ^,^ ^21^ ^,^ ^25^. The presence of lymphocytes is common in these cases, characterizing the inflammatory process^8^ ^,^ ^21^. This was confirmed in our study because an inflammatory infiltrate with predominance of lymphocytes and the presence of necrotic areas were observed in the bleached group at 24 h. The bleached group at 10 days presented a greater number of lymphocytes, characterizing a chronic inflammatory process and necrosis. In both groups (bleached 24 h and 10 days groups), the predominant score was 3 (medium), i.e. moderate inflammatory infiltrate and a score of 4 with presence of necrosis areas. Therefore, our hypothesis that there would be a severe inflammatory process after bleaching with 38% HP was confirmed after 10 days of tooth bleaching. In fact, the presence of necrosis is characteristic of severe damage^6^. The necrosis process reduces cell viability, leading to cell death. This specific type of cell death causes intense damage *in vivo* because high amounts of intracellular components (including enzymes) are released, causing irreversible damage to cells^21^.

Previous *in vivo* studies showed pulpal responses to a single bleaching process ranging from a mild inflammatory reaction to acute inflammation, or even partial necrosis of the coronal pulp tissue^9^ ^,^ ^21^. In addition, there is evidence that repetitive bleaching with 35% HP may lead to morphologic and specific elemental changes and decrease the calcium ion concentration^6^ ^,^ ^20^. By using scanning electron microscopy, we observed significant changes in the prismatic structure of enamel after consecutive applications of HP^6^ ^,^ ^20^. For this reason, we performed two bleaching sessions with a 7-day interval between them, and each session consisted of two 15-min applications of 38% HP; a common situation in clinical practice. The manufacturer of the product suggests an interval of 3 to 5 days between sessions. In our study, moderate and intense inflammatory infiltrate and necrotic areas were present in the pulp even 10 days after the last session, so we can suggest that this interval between sessions is insufficient for an effective pulp response, and that this may increase the damage to the pulp.

The presence of healthy pulp in teeth after a bleaching procedure in some studies^7^ ^,^ ^9^ can be explained by the analysis time of the dental elements after the last bleaching session. In our study, we evaluated the teeth after 24 h and after 10 days, respectively. In the second group (after 10 days), cells which are characteristic of the chronic inflammatory process and angiogenesis were observed, and were associated with reactivity to *ll-1β*, *TNF-β*, *FGF-2* and osteocalcin. This fact suggests that 10 days after the last bleaching session there was inflammation, tissue repair and antioxidant defense because of cell exposure to oxygen from the degradation of HP. Therefore, some cell responses were only evident after the procedure.

In our study, there was immunoreactivity to *GPX*, which also acts as an antioxidant in defense of cells exposed to H_2_O_2_ ^10^, attesting to the arguments abovementioned about the release and beneficial effects of antioxidant agents *in vivo*. In fact, *GPX* is an intracellular antioxidant enzyme that enzymatically reduces H_2_O_2_ to water to limit its harmful effects^10^. Since hydrogen peroxide remains trapped in dental structures even after the gel is removed from the enamel^4^, oxygen ions can activate the production of GPX, which was present in the pulp after 10 days of bleaching.

*ll-1β* is a proinflammatory cytokine and is highly produced in structural cells from pulp tissue such as fibroblasts, odontoblasts, and mesenchymal stem cells, along with immune cells when they are exposed to bacterial and dental materials^8^ ^,^ ^21^. *TNF-β* is a product of activated leukocytes and is another proinflammatory mediator. It is commonly produced in the inflamed dental pulp, with a significant increase in reversible stages of pulp inflammation^1^ ^,^ ^16^. Both are powerful modulators of bone resorption and inhibitors of collagen production^16^. Depending on the concentration of *ll-1β* in the pulp, this cytokine may either have a regenerative or degenerative effect on tissue^21^. There is evidence that bleaching with HP increases collagen degradation in dentin and even in gastric mucosa^14^ ^,^ ^15^ ^,^ ^24^. In addition, important changes in the prismatic structure and biochemical properties of the enamel have been previously observed, such as the loss of carbonate and proteins from the enamel and dentin along with an increase in proteolytic activity and a reduction of collagen^4^ ^,^ ^20^. Accordingly, this may increase the diffusion channels and tissue permeability, thus enhancing pulp damage^6^. In our study, both cytokines abovementioned were produced in the bleached groups. Immunohistochemistry studies suggest that odontoblasts are not only capable of initiating the immune response of the pulp to invasive bacteria by increasing the production of *ll-1β* (for example), but also limiting its intensity^8^ ^,^ ^21^. Odontoblasts produce osteocalcin, which induced a pattern of healing similar to that of *FGF-2* in an *in vivo* model of angiogenesis, and played a role in the regulation of dental pulp repair in reversible pulpitis^1^. In our study, we observe the presence of osteocalcin in the periphery of the inflamed pulp tissue with confocal photomicrography in the bleached group at 24 h because it is a marker of odontoblast differentiation^1^ ^,^ ^21^. In the bleached group at 10 days, the reactivity to osteocalcin was significantly higher in comparison with the other groups; this leads to the conclusion that odontoblasts are stimulated to release this protein after the clinical procedure, what may indicate repair and healing in the tissue in an attempt to limit the immune response and the inflammatory process.

*FGF-2* is also an angiogenic marker with similar action to osteocalcin, being fundamental for pulp repair in response to injury, and plays an important role in mineralization^1^ ^,^ ^19^. Like osteocalcin, the production of *FGF-2* is increased in pulp with a reversible inflammatory process, which in turn leads to a higher occurrence of fibrosis and calcification^1^. *FGF-2* was produced in both bleached groups, and osteocalcin was present in large quantities 10 days after the clinical procedure, which may suggest a greater possibility of pulpal fibrosis and calcification because the role of the two mediators.

Attempts to extrapolate these results directly to humans should be made with caution since rat teeth are not exactly similar to human teeth, especially regarding dentine thickness. However, studies have indicated that topical treatment with HP can lead to an inflammatory process, tissue repair, and necrosis under clinical conditions where dentine is very thin^9^. There is also evidence of coagulation necrosis occurring in the coronal pulp, and deposition of reactive dentin in the radicular pulp of bleached incisors with irreversible damage, and to the detriment of any reaction to the same procedure in premolars which have thicker enamel and dentin^7^ ^,^ ^9^. This clarifies the situation, because the intensity of the pulp response is inversely related to the enamel and dentin thickness; important structures in the protection of the pulp tissue against toxic products released from dental materials^4^ ^,^ ^25^.

## Conclusion

Tooth bleaching with 38% HP in rats causes moderate pulp inflammation after 24 h, and severe inflammation with necrotic areas after 10 days. However, there was the presence of markers that are related to pulp tissue repair.
